# Optimal presale strategy under the deposit inflation mode: Ordinary or sinking strategy

**DOI:** 10.1371/journal.pone.0315275

**Published:** 2025-03-26

**Authors:** Weier Situ, Yongqing Zhang, Xiting Lyu, Wei Zhang

**Affiliations:** School of Business, the University of Shanghai for Science and Technology, Shanghai, China; Xidian University, CHINA

## Abstract

In the context of the rapid development of e-commerce, the advanced selling strategy has become an important means widely used by online retailers, and the innovation of this advanced selling strategy has been extended to the logistics service level. Based on the consumer utility theory, combined with the strategic behavior of consumers, this paper compares the advantages and disadvantages of ordinary pre-sale strategy (OPS) and sinking pre-sale strategy (SPS) under the deposit inflation mode. The research results show that when consumers have a high level of awareness of logistics services and a moderate proportion of tail payment, the sunken pre-sale strategy has more advantages, and the improvement of consumers’ perception coefficient of logistics, proportion of tail payment, and SPS logistics speed is conducive to the increase of retailers’ profits. In addition, retailers need to be cautious when setting pre-sale prices and deposits to avoid excessive deposit settings leading to the loss of consumers. Regardless of the inflation factor of the deposit, the amount of payment remains unchanged, but the higher coefficient helps to increase demand and profit. The research not only provides theoretical support for retailers in formulating advanced selling strategies but also reveals the close relationship between consumer behavior and the market.

## 1. Introduction

In recent years, with the increasingly fierce competition in the electronic commerce industry, advance selling has become one of the most common marketing methods of online retail [1–2], especially in large e-commerce shopping carnivals. The “deposit + final payment” presale format is popular and widely used by retailers. This presale model first emerged during the 2017 Singles’ Day shopping extravaganza. Tmall adopted this model, ultimately driving transaction volume to a record high of 168.2 billion RMB. Following the success of Tmall’s pre-sale model, other e-commerce platforms quickly followed suit. By the 2019 Singles’ Day shopping festival, pre-sale revenue had already accounted for 30% of the total sales [3]. In 2021, JD.com saw a year-on-year increase of 640% in pre-sale orders and a 437% increase in the quantity of goods sold [4]. Consumers pay a deposit in the advance selling period, wait a few days, and then give the final payment during the regular selling period. Through the deposit presale strategy, retailers can target consumers in advance and use this to accurately forecast consumer demand, thus reducing inventory risk. Moreover, it can obtain pre-purchase funds early to relieve the financial pressure [5–6]. Consumers lock in goods in advance to avoid the risk of out-of-stock during the regular selling period. And sometimes they can receive additional discounts by advance orders [7–8]. However, this way of sale also has certain drawbacks, such as longer waiting times during the advance selling period [9] and uncertainty of product valuation [10–11]. In response, e-commerce companies have also proposed several countermeasures to improve and optimize the presale strategy. Not only do they give greater discounts on prices, such as the deposit inflation model, but they also innovate in the logistics and delivery, such as the sinking presale strategy (SPS). SPS means that after the customer pays the deposit, retailers will store the customer’s ordered products in advance to the nearest front warehouse and deliver the packages as soon as the customer pays the balance [3], where the sinking presale strategy is adopted, logistics transportation is referred to as sinking logistics. Compared to the ordinary presale strategy (OPS), the SPS delivers products to consumers in a faster and more flexible way. Nowadays, e-commerce competition is not only a price war but also a logistics war [12–13].

Deposit-inflating presale strategy (DPS) is one of the most popular promotional strategies by online retailers and consumers in recent years. Deposit inflation means that the deposits paid by consumers in booking products are inflated by a certain factor to enjoy discounts when paying the final price. For example, if the deposit for a product is $20 and the deposit-inflating factor is 1.5, the deposit of $20 can be inflated and offset against the final payment of $30 ($20 * 1.5), of which the discount is $10. Due to its deposit inflation effect, the DPS makes the consumer’s perceived value higher, which attracts more consumers to pay the deposit during the advance selling period. Thus, it has become one of the retailers most widely used promotional strategies.

The deposit-inflating presale strategy is a concession to consumers in terms of price promotion. As for the innovation in logistics service, SPS was launched in 2019 as a new pre-sale model for e-commerce platforms. SPS greatly shortens the previous logistics time and reduces the risk of delivery slowdown due to the blockage of express transit stations. More importantly, it enhances the shopping experience of consumers, thus making the product repurchase rate significantly higher [13]. However, the SPS needs to invest and build the warehouse in advance. The front-end investment is large, and the warehouse operation and maintenance also cost a certain amount of money. In addition, if consumers do not pay the final payment, the logistics cost of early delivery and product recall will be borne by the retailers themselves. Since the SPS has not been created for a long time and is not widely adopted, many consumers’ perception of this logistics method is not obvious [3]. Therefore, retailers need to make decisions on whether to adopt the new type of sinking presale strategy.

To solve the problem of retailers’ decision of the presale strategy, this paper compares the OPS and the SPS under the deposit inflation mode, considering the strategic consumers and the uncertainty of valuing presale products. Then it analyzes the influence of each parameter on presale pricing, consumer demand, and retailer’s profit from three perspectives of consumer behavior, presale deposit, and sinking logistics. The paper provides instructive guidance for retailers when utilizing the presale strategy in their marketing practices. The main contributions of this paper are as follows: Firstly, based on consumer utility theory, the paper considers consumer strategic behavior in presales. It constructs game models for ordinary presale strategy and sinking presale strategy under a savings-inflation mode, and solves for their optimal decisions. This enriches theoretical research on how consumers perceive the value of presale products and make purchasing decisions. Secondly, the study emphasizes the importance of setting presale prices and deposit amounts reasonably, as well as considering factors such as logistics perception, final payment ratio, and sinking logistics speed when choosing presale strategies. Finally, from the perspectives of consumer behavior, prepaid deposits, and sinking logistics, the research compares and discusses the effects of various parameters on presale pricing, consumer demand, and profit under sinking presale strategy and ordinary presale strategy. This helps understand consumer behaviors and preferences, providing insights for retailers to formulate presale strategies and effectively participate in market competition.

The remainder of this article is organized as follows. In Section 2, we present the literature review. Section 3 describes the problem and sets forth the research hypotheses. In Sections 4 and 5, we solve the baseline model OPS and the comparison model SPS separately to obtain the optimal presale price, demand, and profit. A comparison of two presale strategies is performed in Section 6. Numerical analysis is in Section 7. Finally, Section 8 summarizes the findings of this paper and directions for further research.

## 2. Literature reviews

For the sake of clarity, we review the related literatures from the perspectives of these two aspects: strategic consumer behavior in advance selling and retailers’ presale strategies for higher profits.

### 2.1. Strategic consumer behavior in advance selling

In the field of advance selling research, strategic consumer behavior has attracted increasing attention from researchers. It refers to the consumer behavior that consumers will choose the timing of purchase more rationally and wait for further price reduction of the product based on their expectation of the future price of the product [14]. For example, Tian and Wang [15] assumed that there are two types of customers in the market. In the advance selling period, the high-type consumers arrive and their valuation is deterministic. In the regular selling period, the low-type consumers arrive, who have strategic behavior, and their valuation depends on the preorder outcome. Peng et al. [16] also divided consumers into these two types. And they further studied the price guarantee policies of a seller practicing the presale strategy under the social learning. Zhang et al. [17] investigated the impact of consumers’ anticipated regret, such as action regret and inaction regret, on the mixed bundle strategy of presale under the uncertain consumer evaluations. They found that consumer regret has a significant impact on retailers and suggested that retailers can bundle two products with large price differences to reduce the negative impact of consumer regret to maximize profits. The literatures showed that consumer risk aversion [18], regret behavior [19], anxiety [20] and disappointment aversion [21] also act on consumers’ strategic behavior and greatly influence pre-sale purchases. Liu et al. [22] sheded light on the influence mechanism of anchoring and the payment pain reduction effect on consumer behaviour and online product pricing.

In addition to intrinsic consumer factors, the influence of external factors on consumers’ strategic behavior can also play a role in retailers’ decisions. Wang et al. [23] proposed a novel way to measure consumer utility based on consumer’s time preference for the presale lead time. They found that the two-stage presale strategies are more beneficial for the seller in maximizing its revenue. When the presale lead time is long or the retail price is high, the retailer should set a high deposit. Based on the time preference, Jiang et al. [24] investigated three presale models for innovative products, namely, the no-presale model, the manufacturer presale model and the retailer presale model. Zhou et al. [25] considered the arrival process of consumers and developed a model of retailers’ time decisions with and without presale strategy. Liu et al. [26], Ji et al. [27] and Wang et al. [28] found that consumers have a reference price effect on pre-sale and on-sale prices, or on the prices of different retailers, and investigated the impact of the reference price effect on retailers’ presale strategies. Sun et al. [29] explored the role of online reviews on retailers’ pricing strategies by analyzing the effect of consumer reviews in the advance selling period on consumer strategy behavior in the regular selling period. They found that if a loyal consumer chooses to wait until the normal sales period to purchase, the retailer will resort to lowering the presale price to induce consumer reviews and stimulate consumer demand in the regular selling period.

Existing literature has provided a more comprehensive study of strategic consumer behavior in advance selling. Based on these, our paper provides a more detailed classification of consumer behavior. First, consumers are classified into strategic and short-sighted consumers according to their valuation. Short-sighted consumers purchase only during the advance selling period. Strategic consumers compare the valuation between the advance selling period and the regular selling period, and make purchases based on merit. Then, we subdivide the consumers, who pay the deposit in the advance selling period, into two categories. One is to pay the final payment and the other is not to pay the final payment. This assumption is more in line with life and has a more realistic basis.

### 2.2. Retailers’ presale strategies for higher profits

There is a wide variety of presale strategies. According to the payment stage, it can be divided into the single-stage presale which is a one-time full payment, and the two-stage presale which includes deposit and final payment. It can also be divided into discounted presale, equal presale, and premium presale according to the presale price. Nowadays, presale strategies are often accompanied by promotions such as deposit inflation, price reduction and giveaways. It is critical for retailers to choose the optimal presale strategy.

Consumers’ payment methods and retailers’ advance selling models have a significant impact on purchase behavior and pricing decisions. Liu et al. [30] consider the impact of payment pain passivation effect on consumer psychology, and explore the pricing decision of online products under deposit appreciation. The results show that discount advance selling is not necessarily the optimal choice strategy. Based on the situation that the uncertainty of product value in the advance selling period induces consumers’ strategic behavior, Yin and Wang [31] studied how to use the deposit appreciation to eliminate its negative impact. The results show that the value-added coefficient and the proportion of deposit payment in the customized value-added strategy significantly affect the consumer’s purchase behavior, and the appropriate decision can effectively alleviate the consumer’s delayed purchase behavior. Zhang et al. [32] investigated the effect of deposit expansion, consumer’s valuation changes and return policy on the retailer’s presale strategy. They found that the retailer gains further profit under the presale strategy with a deposit, due to the reduction of inventory risk and exploitation of consumer’s valuation uncertainty. Considering the purchasing behavior of risk-averse strategic consumers, Xu and Dong et al. [33].examined the optimal order quantity and presale price of retailers under discounted and deposit-inflated presale modes. They found that for strategic consumers, the optimal presale price under the discounted presale model is smaller than that under the “deposit inflation” presale, but the optimal presale order quantity under the discounted presale is larger than that under the “deposit inflation” presale model. In another paper, they further studied the wholesale ordering and option combination ordering strategies of retailers under the above two pre-sale models [34].

Due to the short years of the sinking presale face, there are not many related studies and only very few scholars have explored it. Liu et al. [35] considering the influence of sinking advance selling strategy and risk aversion behavior on the decision-making of logistics integrators, discussed the decision-making of logistics integrators under different discount advance selling strategies. The study found that high discount advance selling strategy can eliminate the influence of uncertain demand risk, while low discount advance selling strategy cannot. The research of Chen et al. [36] also discussed the impact of decision makers’ risk aversion on the joint decision of optimal transportation volume and product pricing in a new sinking logistics model, and further compared the carbon emissions of traditional logistics mode and new logistics mode. Wang et al. [37] studied retailers’ promotional strategies and delivery strategies with or without early delivery in a deposit pre-sale model. They found that even if the cost of returning goods is higher than the deposit, the SPS always outperforms on-time delivery as long as the percentage of consumers paying the final payment is high enough. Zhang et al. [38] also studied the strategic competition of SPS between two e-commerce platforms with asymmetric logistics services, and considered consumers with bounded rationality. They found that only when the number of bounded rational consumers is below a certain threshold, the two merchants will adopt SPS at the same time. Different from previous studies, they considered the differences in logistics services between OPS and SPS, and included logistics factors into the study. Although the above studies discuss the sinking logistics model, they do not consider the logistics perception of consumers on the sinking logistics transportation mode. At the same time, considering the impact of discounts on decision makers, but this study considers the decision-making situation under the influence of deposit expansion.

In summary, the sinking pre-sale strategy is usually applicable to the two-stage pre-sale model of deposit +  end-of-period payment. However, there is no research on SPS in the deposit expansion mode. Therefore, based on the strategic behavior of consumers, this paper constructs and compares two pre-sale strategies under the deposit inflation mode-the sunk pre-sale strategy and the ordinary pre-sale strategy, introduces the consumer ‘s logistics perception factors and discusses the retail pre-sale pricing strategy. The main research questions of this paper are:

How does consumer strategic behavior affect consumer purchase decision and retailer’s advance selling strategy under deposit inflation mode?
Considering the utility of consumers, which advance selling strategy is the optimal decision?
What are the key factors affecting the retailer’s advance selling strategy choice? How do these factors affect their decision-making?

Unlike existing literature that primarily studies deposit inflation and discount pre-sale models, this paper provides a comprehensive analysis of the new sinking pre-sale model for the first time, considering both pricing and logistics factors. While this study shares the same objective as previous research—exploring optimal strategies for retailers to enhance profits and meet demand—the difference lies in the new sinking pre-sale model, which reduces delivery times and improves logistics efficiency through pre-positioned inventory. The emergence of these differences in models may be attributed to advancements in modern logistics technology and the use of predictive analytics, enabling the new strategy to respond more effectively to changes in demand and enhance operational efficiency.

## 3. Problem description and assumption

### 3.1. Problem description

**Fig 1:**
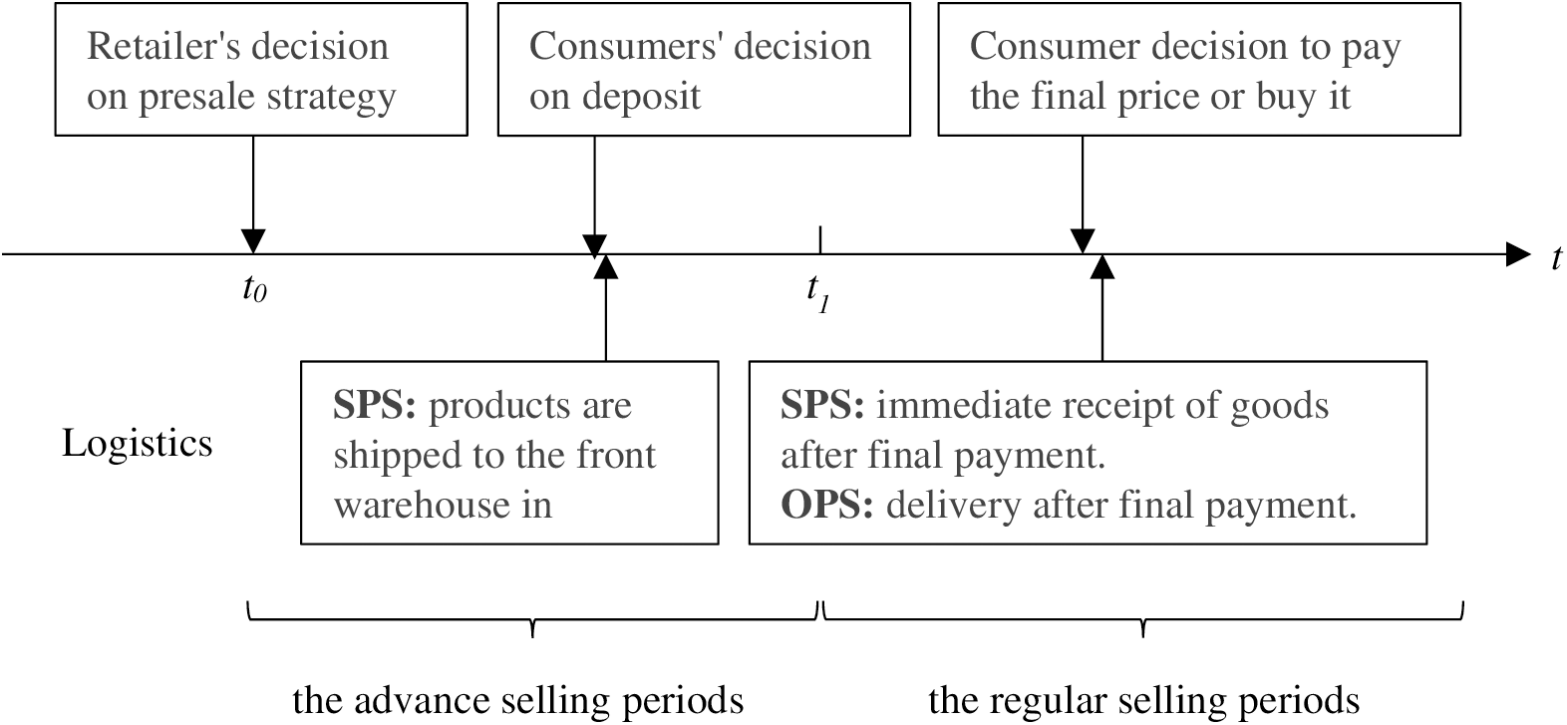
Sequence of events for presale strategies.

The paper assumes a situation where there is only one monopolistic retailer in the market, selling a product through the deposit-inflated presale. The entire sales process is divided into two periods: the advance selling period and the regular selling period. The retailer first announces the presale format, including the deposit amount, the inflation factor, the prices of presale and on sale, and the logistics method. During the advance selling period, consumers decide based on the information announced whether to pay a deposit to lock in the product. In the regular selling period, consumers who have not made a deposit can make a full payment during this period, and consumers who have paid a deposit can choose whether to pay the final payment. The sequence of events is shown in Fig 1.

### 3.2. Assumptions

For retailers, the primary concern is how to design presale strategies to maximize profits. For consumers, the primary concern is how to design decision-making processes to maximize product utility. In order to address this issue, we have constructed a fundamental model and put forward our assumptions. The relevant parameters and hypotheses are as follows:

Assumption 1. In this model, we imagine that there are several consumers *N* in the market, and each consumer buys at most one product. The consumers are divided into two categories: strategic consumers and short-sighted consumers. The proportion of strategic consumers is assumed to be *α*, and the proportion of short-sighted consumers is assumed to be 1−α. Both types of consumers arrive at the market during the advance selling period.

Assumption 2. Short-sighted consumers will only make decisions during the advance selling period and buy the product when the utility gained from purchasing the product is non-negative. Strategic consumers place orders in the advance selling period when the utility of presale is non-negative and greater than the utility of in-stock purchases.

Assumption 3. Due to the uncertainty of presales, consumers who pay a deposit during the advance selling period reevaluate the utility they receive during the regular selling period, so some consumers opt out or do not pay the final payment on time. Assume that the percentage of consumers who choose to pay the final payment is *β*, and the percentage of consumers who do not pay the final payment is 1−β.

Assumption 4. The retailer has two presale strategies to choose from the ordinary presale strategy and the sinking presale strategy. Assume that retailers make decisions based on profit maximization, and the product unit cost in the OPS or the regular selling period in the SPS is $c_{o}$, including the production cost and the logistics cost. The product unit cost under the advance selling period in the SPS is assumed to be $c_{s}=c_{o}+\frac{\delta }{l}$ [3,39]. *l* is the logistics transportation time of SPS relative to OPS, and *δ* is the logistics cost factor.

Assumption 5. We assume that the faster the logistics speed, the higher the logistics cost. Moreover, if the consumer does not pay the final payment in the sinking presale strategy, the retailer needs to recall the shipped product, so that the cost of recalling the product is $c_{b}=\frac{\gamma }{l}$. *γ* is the logistics recall cost factor, and γ>δ.

Assumption 6. The retailer uses the pricing strategy of price commitment and deposit inflation. The price commitment indicates that the retailer informs consumers in advance of the presale rules, announces the pre-sale price and the on-sale price, and does not change the prices once they are announced. This way gives consumers the right to be fully informed.

Assumption 7. The on-sale price *p* is determined by the market, while the pre-sale price is decided by the retailer, and a percentage of the amount is set as a deposit for pre-order the product. The deposit is assumed to be $f=k\cdot p_{a}$, where *k* is the deposit percentage. The retailer offers a certain price discount to consumers in the form of an inflated deposit. The deposit is increased by a deposit inflation factor *θ*, θ≥1. When θ=1, it means that the retailer does not offer a discount, and θ>1 means that the consumer offers a discount $\left(\theta -1\right)f$. At this point, the amount paid for the product is $p_{m}=p_{a}-(\theta -1)\cdot k\cdot p_{a}$. All Symbol descriptions are shown in Table 1.

**Table 1:** Description of relevant symbols.

Symbol	Description
$U_{i}$	The total utility of the product purchased by the consumer, where i=o,a,s, *a* denotes the advance selling period for normal presale, *s* denotes the advance selling period for sinking presale, and *o* denotes the regular selling period of the two presale strategies
*v*	Consumer valuation of the product, obeying the Uniform Distribution of [h_1_, h_2_]
*λ*	Consumer’s perceived utility coefficient for presale products, 0≤λ≤1
*N*	The number of potential consumers in the market
*α*	The proportion of strategic consumers
*β*	The proportion of consumers who pay the final price
*p*	The price of the product in the regular selling period
$p_{a}$	The price of the product in the advance selling period
$p_{m}$	The actual price paid by the consumer, $p_{m}=p_{a}-(\theta -1)\cdot k\cdot p_{a}$
*f*	The deposit in the advance selling period
*k*	The deposit as a percentage of the pre-sale price
*θ*	The deposit inflation factor
*l*	The logistics transportation time when using sinking pre-sale strategy
*η*	Consumer’s logistics perception factor for sinking presale strategy
$c_{o}$	The cost of products cost when adopting normal pre-sales
$c_{s}$	The cost of products when adopting sinking presale, $c_{s}=c_{o}+\delta /l$
$c_{b}$	The cost of recalling products, $c_{b}=\gamma /l$
$Q_{i}$	Demand
*π*	Profit

## 4. Benchmark model: Ordinary presale strategy

For the ordinary presale strategy, the consumer’s utility function consists of the product valuation and the out-of-pocket price. Considering the uncertainty of the pre-sale product, the riskiness of paying in advance, and the waiting time of the advance selling period, there is a loss in the consumer’s valuation of the product [21,36]. Let *λ* be the product valuation factor of the consumer in the advance selling period. Therefore, the utility function obtained by short-sighted and strategic consumers who purchase the product during the advance selling period of the OPS is


$$U_{a}=\lambda v-\left[p_{a}-\left(\theta -1\right)f\right]$$ (1)

Short-sighted consumers ignore the regular selling period. However, strategic consumers evaluate the utility of both periods. The utility function obtained by a strategic consumer who buys the product in the regular selling period is:


$$U_{o}=v-p$$ (2)

For short-sighted consumers, they will choose to buy in the advance selling period if $U_{a}\geq 0$, otherwise withdraw from the market. By solving the inequality $U_{a}\geq 0$, we can obtain the valuation range $v\geq \frac{\left[1-\left(\theta -1\right)k\right]p_{a}}{\lambda }$. Let $v_{1}=\frac{\left[1-(\theta -1)k\right]p_{a}}{\lambda }$, so that the number of short-sighted consumers who pay a deposit during the advance selling period is:


$$Q_{11}=\left(1-\alpha \right)N\cdot \frac{h_{2}-v_{1}}{h_{2}-h_{1}}=\frac{N\left(1-\alpha \right)\left[\lambda h_{2}-\left(1-(\theta -1)k\right)p_{a}\right]}{\lambda \left(h_{2}-h_{1}\right)}$$ (3)

For strategic consumers, if $U_{a}\geq U_{o}$ and $U_{a}\geq 0$, they choose to buy in the advance selling period. Solving the two inequalities, we get $v\leq \frac{p-\left[1-(\theta -1)k\right]p_{a}}{1-\lambda }$ and $v\geq \frac{\left[1-(\theta -1)k\right]p_{a}}{\lambda }=v_{1}$ Let $v_{2}=\frac{p-\left[1-(\theta -1)k\right]p_{a}}{1-\lambda }$. For the demand of strategic consumers to exist in this case, it is necessary to satisfy $v_{1}\leq v_{2}$. It means $p_{a}\leq \frac{\lambda p}{1-\left(\theta -1\right)k}$. At this point, the number of strategic consumers who pay a deposit in the advance selling period is:


$$Q_{12}=\alpha N\cdot \frac{v_{2}-v_{1}}{h_{2}-h_{1}}=\frac{\alpha N\left[\lambda p-\left(1-(\theta -1)k\right)p_{a}\right]}{\lambda \left(1-\lambda \right)\left(h_{2}-h_{1}\right)}$$ (4)

Strategy consumers who decide to wait until the regular selling period, choose to buy the product when $U_{o}\geq 0$. So, we get $v_{3}=p$. Knowing that condition $p_{a}\leq \frac{\lambda p}{1-\left(\theta -1\right)k}$ is satisfied, it can be deduced that $v_{1}\leq v_{3}\leq v_{2}$. Therefore, the number of strategic consumers who buy the product in the regular selling period is:


$$Q_{13}=\alpha N\cdot \frac{h_{2}-v_{2}}{h_{2}-h_{1}}=\frac{\alpha N\left[\left(1-\lambda \right)h_{2}-p+\left(1-(\theta -1)k\right)p_{a}\right]}{\left(1-\lambda \right)\left(h_{2}-h_{1}\right)}$$ (5)

**Fig 2:**
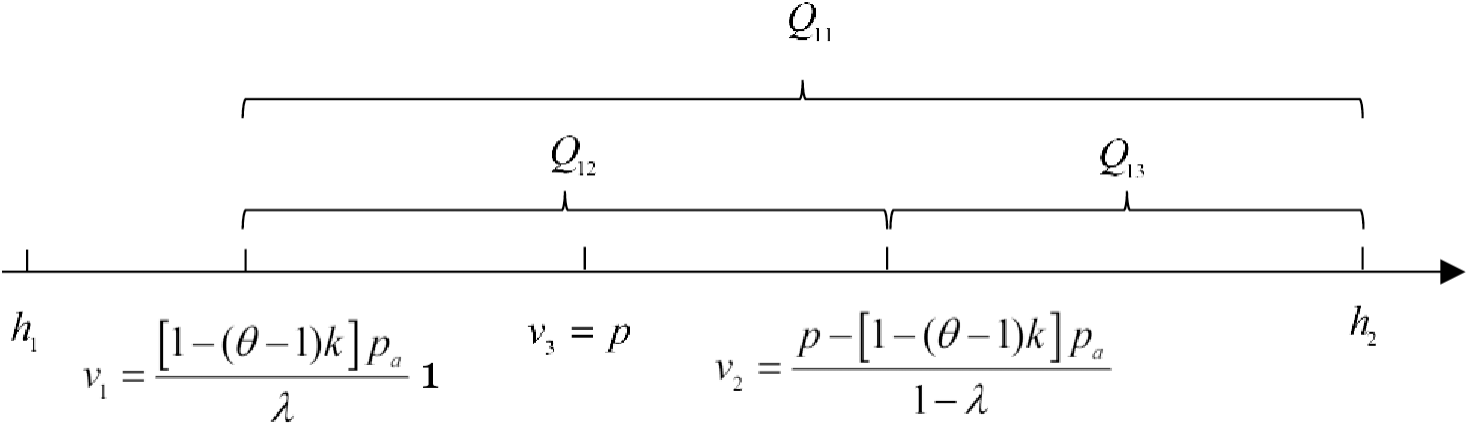
Consumers’ purchase under the ordinary presale strategy.

Consumers’ purchases under the OPS are shown in Fig 2.

Therefore, the consumer demand function and profit function in the OPS are:


$$D_{1}=Q_{11}+Q_{12}+Q_{13}=\frac{N}{\lambda \left(h_{2}-h_{1}\right)}.\left[\lambda h_{2}-\left(1-(\theta -1)k\right)p_{a}\right]$$ (6)

$$\pi_{1}=\beta \left(Q_{1}+Q_{1}\right)\left(p_{a}-\left(\theta -1\right)f-c_{o}\right)+Q_{2}\left(p-c_{o}\right)+\left(1-\beta \right)\left(Q_{1}+Q_{1}\right)f$$ (7)

In the profit function, the first term represents the profit from a successful purchase during the advance selling period, the second term represents the profit during the regular selling period, and the third term represents the profit obtained by foregoing the final payment during the advance selling period. The third item represents the deposit received by the retailer when the consumer waives the final payment.

Then, we solve the model to obtain the optimal solution.

Eq. (7) is a quadratic function of profit $\pi_{1}$ with respect to the presale price $p_{a}$, so the first-order and second-order derivatives of $\pi_{1}$ with respect to $p_{a}$ are found separately for:


$$\frac{d\pi_{1}}{dp_{a}}=\frac{N}{\lambda \left(1-\lambda \right)\left(h_{2}-h_{1}\right)}\cdot \left\{\begin{array}{l} \left(1-\alpha \right)\left(1-\lambda \right)\left(k+\beta -k\beta \theta \right)\lambda h_{2}+\left(k+\beta -k\beta \theta \right)\alpha \lambda p\\ +\left(1-(\theta -1)k\right)\left[\left(1-\alpha \right)\left(1-\lambda \right)+\alpha \right]\beta c_{o}+\left(1-(\theta -1)k\right)\alpha \lambda \left(p-c_{o}\right)\\ -2\left(k+\beta -k\beta \theta \right)\left[1-(\theta -1)k\right]\left[\left(1-\alpha \right)\left(1-\lambda \right)+\alpha \right]p_{a} \end{array}\right\}$$ (8)

$$\frac{d^{2}\pi_{1}}{dp_{a}{}^{2}}=-2\frac{N}{\lambda \left(1-\lambda \right)\left(h_{2}-h_{1}\right)}\cdot \left(k+\beta -k\beta \theta \right)\left[1-(\theta -1)k\right]\left[\left(1-\alpha \right)\left(1-\lambda \right)+\alpha \right]<0$$ (9)

Since $\frac{d^{2}\pi_{1}}{dp_{a}{}^{2}}<0$, there is a unique optimal solution $p_{a}{}^{*}$ that maximizes $\pi_{1}$. Solving the equation $\frac{d\pi_{1}}{dp_{a}}=0$, and substituting $p_{a1}{}^{*}$ into Eqs. (6-7), the optimal solutions can be obtained as follows:


$$p_{a1}{}^{*}=\frac{B\left(1-\alpha \right)\left(1-\lambda \right)\lambda h_{2}+\alpha B\lambda p+AE\beta c_{o}+A\alpha \lambda (p-c_{o})}{2ABE}$$ (10)

$$D_{1}{}^{*}=\frac{N}{2BE\lambda \left(h_{2}-h_{1}\right)}.\left[B(E+\alpha )\lambda h_{2}-\alpha B\lambda p-AE\beta c_{o}-A\alpha \lambda (p-c_{o})\right]$$ (11)

$$\pi_{1}{}^{*}=\frac{N}{4ABE\lambda (1-\lambda )h_{2}}\left\{\begin{array}{l} {\left[B\left(1-\alpha \right)\left(1-\lambda \right)\lambda h_{2}+B\alpha \lambda p+A\alpha \lambda (p-c_{o})-AE\beta c_{o}\right]}^{2}\\ +4AE\alpha \lambda (p-c_{o})\left[B\left(1-\lambda \right)h_{2}-Bp+A\beta c_{o}\right] \end{array}\right\}$$ (12)

For writing convenience, let A=1−(θ−1)k, B=k+β−kβθ, $E=\left(1-\alpha \right)\left(1-\lambda \right)+\alpha$.

## 5. Contrast model: Sinking presale strategy

When the retailer adopts a sinking presale strategy, consumers gain additional logistics perception utility $\frac{\eta }{l}$. *η* is the logistics perception factor of sinking presale. The faster the logistics speed or the greater the logistics perception factor, the greater the consumer’s perceived logistics utility. At this point, the consumer’s utility function consists of the product valuation, the out-of-pocket price, and the perceived logistics utility. Therefore, the utility function obtained by short-sighted and strategic consumers who purchase the product during the advance selling period of the SPS is:


$$U_{s}=\lambda v-\left[p_{a}-\left(\theta -1\right)f\right]+\frac{\eta }{l}$$ (13)

The utility function obtained by a strategic consumer who buys the product in the regular selling period is:


$$U_{o}=v-p$$ (14)

For short-sighted consumers, they choose to buy the product in the advance selling period when $U_{s}\geq 0$, otherwise withdraw from the market. Solving for $v\leq v_{4}=\frac{Alp_{a}-\eta }{\lambda l}$. The number of short-sighted consumers who pay a deposit during the advance selling period is:


$$Q_{21}=\left(1-\alpha \right)N\cdot \frac{h_{2}-v_{4}}{h_{2}-h_{1}}=\frac{N\left(1-\alpha \right)\left[\lambda lh_{2}-Alp_{a}+\eta \right]}{\lambda l\left(h_{2}-h_{1}\right)}$$ (15)

For strategic consumers, if $U_{s}\geq U_{o}$ and $U_{s}\geq 0$, they choose to buy in the advance selling period. Solve for $v\leq v_{5}=\frac{lp-Alp_{a}+\eta }{l\left(1-\lambda \right)}$ and $v\geq v_{4}=\frac{Alp_{a}-\eta }{\lambda l}$, respectively. It needs to satisfy the inequality $v_{4}\leq v_{5}$, and solve for $p_{a}\leq \frac{\lambda lp+\eta }{Al}$. At this point, the number of strategic consumers who pay a deposit during the advance selling period is:


$$Q_{22}=\alpha N\cdot \frac{v_{5}-v_{4}}{h_{2}-h_{1}}=\frac{\alpha N\left[\lambda lp-lAp_{a}+\eta \right]}{l\lambda \left(1-\lambda \right)\left(h_{2}-h_{1}\right)}$$ (16)

Strategy consumers who decide to wait until the regular selling period, choose to buy the product when $U_{o}\geq 0$. So, we get $v\geq v_{3}=p$. Knowing that condition $p_{a}\leq \frac{\lambda lp+\eta }{Al}$ is satisfied, it can be deduced that $v_{4}\leq v_{3}\leq v_{5}$. Therefore, the number of strategic consumers who buy the product in the regular selling period is:


$$Q_{23}=\alpha N\cdot \frac{h_{2}-v_{5}}{h_{2}-h_{1}}=\frac{\alpha N\left[\left(1-\lambda \right)lh_{2}-lp+Alp_{a}-\eta \right]}{l\left(1-\lambda \right)\left(h_{2}-h_{1}\right)}$$ (17)

**Fig 3:**
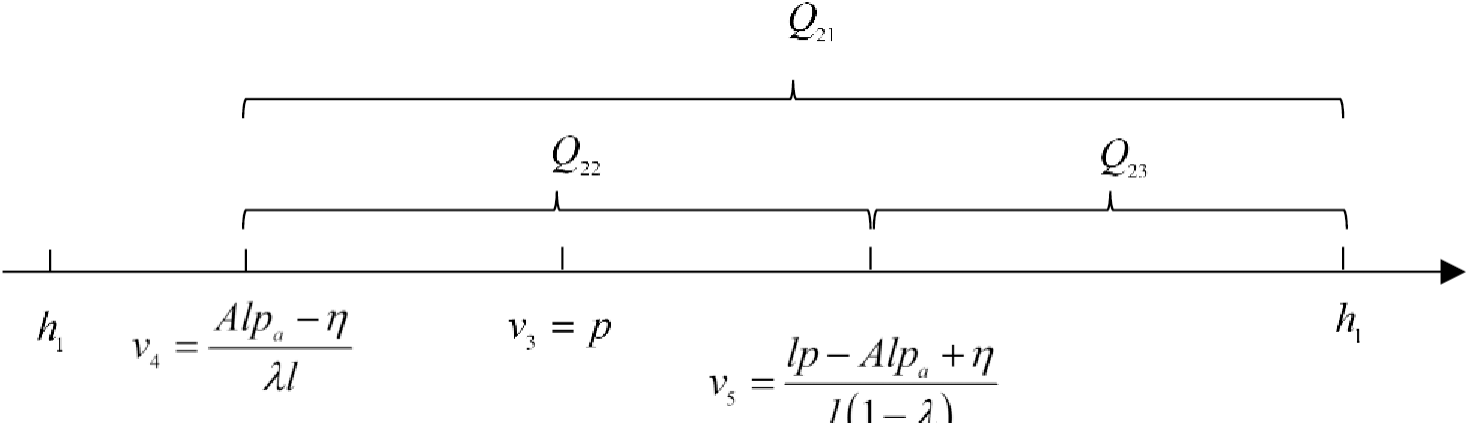
Consumers’ purchases under the sinking presale strategy.

The purchases of consumers under the SPS are shown in Fig 3.

In summary, the consumer demand function and profit function in the SPS are:


$$D_{2}=Q_{21}+Q_{22}+Q_{23}=\frac{N}{\lambda \left(h_{2}-h_{1}\right)}.\left[\lambda h_{2}-Ap_{a}+\frac{\eta }{l}\right]$$ (18)

$$\pi_{2}=\beta \left(Q_{21}+Q_{22}\right)\left(p_{a}-\left(\theta -1\right)f-c_{s}\right)+Q_{2}\left(p-c_{o}\right)+\left(1-\beta \right)\left(Q_{21}+Q_{22}\right)\left(f-c_{b}\right)$$ (19)

The solution procedure is the same as benchmark model. The first-order and second-order derivatives of $\pi_{2}$ with respect to $p_{a}$ are found separately for:


$$\frac{d\pi_{2}}{dp_{a}}=\frac{N}{\lambda \left(1-\lambda \right)\left(h_{2}-h_{1}\right)}\cdot \left\{\begin{array}{l} \left(1-\alpha \right)\left(1-\lambda \right)B\lambda lh_{2}+\left(A+B\right)\alpha \lambda lp+AE\beta c_{o}\\ -A\alpha \lambda lc_{o}+BE\eta +AE\left[\delta \beta +\gamma \left(1-\beta \right)\right]-2ABElp_{a} \end{array}\right\}$$ (20)

$$\frac{d^{2}\pi_{2}}{dp_{a}{}^{2}}=-2\frac{N}{\lambda \left(1-\lambda \right)\left(h_{2}-h_{1}\right)}\cdot ABEl<0$$ (21)

Since $\frac{d^{2}\pi_{2}}{dp_{a}{}^{2}}<0$, there is a unique optimal solution $p_{a}{}^{*}$ that maximizes $\pi_{2}$. Solving the equation $\frac{d\pi_{2}}{dp_{a}}=0$, and substituting $p_{a2}{}^{*}$ into Eqs. (18-19), the optimal solutions can be obtained as follows. Let $G=\delta \beta +\gamma \left(1-\beta \right)$.


$$p_{a2}{}^{*}=\frac{B\left(1-\alpha \right)\left(1-\lambda \right)\lambda lh_{2}+B\alpha \lambda lp+AE\beta lc_{o}+A\alpha \lambda l(p-c_{o})+BE\eta +AEG}{2ABEl}$$ (22)

$$D_{2}{}^{*}=\frac{N}{2BEl\lambda \left(h_{2}-h_{1}\right)}.\left[B\left(E+\alpha \right)l\lambda h_{2}-B\alpha \lambda lp-AE\beta lc_{o}-A\alpha \lambda l(p-c_{o})+BE\eta -AEG\right]$$ (23)

$$\pi_{2}{}^{*}=\frac{N}{4ABE\lambda (1-\lambda )l^{2}(h_{2}-h_{1})}\left\{\begin{array}{l} {\left[\begin{array}{l} B\left(1-\alpha \right)\left(1-\lambda \right)\lambda lh_{2}+B\alpha \lambda lp+BE\eta \\ +A\alpha \lambda l(p-c_{o})-AE\beta lc_{o}-AEG \end{array}\right]}^{2}\\ +4AE\alpha \lambda l(p-c_{o})\left[B\left(1-\lambda \right)lh_{2}-Blp+A\beta lc_{o}+AG-B\eta \right] \end{array}\right\}$$ (24)

## 6. A comparison of two presale strategies

This section compares the ordinary presale strategy and the sinking presale strategy, analyzes the relationship between presale price, demand and profit under the two strategies, and further explores the choice of presale strategies for retailers under different conditions.

### 6.1. Optimal presale price

**Proposition 1** The optimal presale price under the sinking presale strategy $p_{a2}{}^{*}$ is greater than that under the ordinary presale strategy $p_{a1}{}^{*}$.

Proof: $△p_{a}=p_{a2}{}^{*}-p_{a1}{}^{*}=\frac{\left(k+\beta -k\beta \theta \right)\eta +\left[1-(\theta -1)k\right]\left[\delta \beta +\gamma \left(1-\beta \right)\right]}{2\left[1-(\theta -1)k\right]\left[k+\beta -k\beta \theta \right]l}\geq 0$, so $p_{a2}{}^{*}\geq p_{a1}{}^{*}$.

Proposition 1 shows that retailers set a relatively high advance selling price when choosing SPS. Comparing the advance selling prices of the two strategies, it can be seen that the difference between the two prices is caused by the logistics service under SPS. In this case, the retailer packages the product in advance according to the consumer’s deposit, and transports them to the front and cloud warehouses near the destination through sinking logistics. When the pre-sale period ends and the consumer pays the last amount, the product is delivered to the consumer immediately. Compared with OPS logistics, this delivery method requires additional storage investment, maintenance and operating costs. In addition, if consumers do not pay the final payment, the retailer will have to recall the shipped product, and the logistics cost of the return will be borne by the retailer. The increase in the advance selling price indirectly increases the deposit amount, and consumers may choose to pay the final payment due to the higher cost. This is why retailers will set higher presale prices when implementing a sinking presale strategy. In other words, a higher advance selling price can compensate for the additional logistics costs and product recall costs of sunk advance selling, thereby reducing the loss of consumer uncertainty under sinking advance selling model.

### 6.2. Optimal demand

**Proposition 2** When $\eta \geq \frac{\left[1-(\theta -1)k\right]\left[\delta \beta +\gamma \left(1-\beta \right)\right]}{\left(k+\beta -k\beta \theta \right)}$ or $\beta \geq \frac{\gamma \left[1-(\theta -1)k\right]-k}{(1-k\theta )+(\gamma -\delta )\left[1-(\theta -1)k\right]}$, the optimal demand under the sinking presale strategy $D_{2}{}^{*}$ is greater than that under the ordinary presale strategy $D_{1}{}^{*}$.

Proof: $\begin{array}{c} \Delta D=D_{2}{}^{*}-D_{1}{}^{*}=\frac{N}{2Bl\lambda \left(h_{2}-h_{1}\right)}\left\{\left(k+\beta -k\beta \theta \right)\eta -\left[1-(\theta -1)k\right]\left[\delta \beta +\gamma \left(1-\beta \right)\right]\right\}\\ = \{\begin{array}{l} \geq 0,\eta \geq \frac{\left[1-(\theta -1)k\right]\left[\delta \beta +\gamma \left(1-\beta \right)\right]}{\left(k+\beta -k\beta \theta \right)},or\beta \geq \frac{\gamma \left[1-(\theta -1)k\right]-k}{(1-k\theta )+(\gamma -\delta )\left[1-(\theta -1)k\right]}\\ <0,else \end{array} \end{array}$.

Proposition 2 illustrates that when the consumers’ logistics perception factor *η* or the percentage of consumers paying the final payment for sinking presale *β* is above a certain threshold, the retailer will obtain relatively higher demand when choosing the sinking presale strategy.

Combined with Proposition 1, when the above conditions are met, the optimal demand is actually greater, despite the fact that the price of the SPS is higher than that of the OPS. This suggests that consumers’ perception of the sinking logistics can effectively increase consumers’ utility of purchasing the product, or even offset the negative impact of the higher presale price, which in turn attracts more consumers to make purchases. In addition, the greater the percentage of consumers paying the final payment, i.e., the greater the number of consumers completing the two-stage sale of “deposit + final payment”, the greater the consumer demand.

Therefore, when a retailer adopts the SPS, it needs to strongly promote the logistical advantages of the strategy during the advance selling period, which has the fast delivery of the goods after the final payment has been made. This enhances consumers’ perceptions of the sinking logistics, and increases the consumers’ utility of the products, in turn capturing greater market demand. At the same time, the retailer should remind consumers to make the final payment during the final payment period, such as sending messages, to increase the percentage of consumers paying the final payment and prompt them to complete the whole purchase process.

### 6.3. Optimal profit

**Proposition 3** The optimal profit of the retailer with the sinking presale strategy $\pi_{2}{}^{*}$ is greater than that with the ordinary presale strategy $\pi_{1}{}^{*}$ when the consumer’s perception of the sinking logistics *η* or the percentage of consumers paying the final payment *β* is above a certain threshold.

**Proposition 4** The profit of sinking presale strategy $\pi_{2}{}^{*}$ increases as the speed of logistics *l* decreases.

Proof:

△π=π2*−π1*=NBη−AG4ABλ(1−λ)l2h2−h1BEη−AEG+2lB1−α1−λλh2+Bαλp−Aαλ(p−co)−AEβco=≥0,η≥2lB1−α1−λλh2+Bαλp−Aαλ(p−co)−AEβco−AEGBEorβ≥Aαλ(p−co)+Eβco1−kθ1−α1−λλh2+αλp−k1−kθ<0,elseThe first order derivative of $\pi_{2}{}^{*}$ with respect to *l* gives $\frac{d\pi_{2}{}^{*}}{dl}\leq 0$.

Proposition 3 illustrates the impact of consumers’ perception of sinking logistics *η* and the proportion of consumers paying the final payment *β* on optimal profits $\pi^{*}$. When the consumers’ perception or the proportion is larger, the profit of the SPS is more favorable. Combining Propositions 1 and 2, we can find that in this case, the optimal demand of the SPS is larger than that of the OPS, and the price of the SPS is always higher than that of the OPS. Therefore, when one of the above conditions is satisfied, the retailer will prefer to choose the sinking presale strategy to gain more profit.

Proposition 4 indicates the effect of sinking logistics time *l* on optimal profit $\pi_{2}{}^{*}$. The faster the sinking logistics speed, the greater the optimal profit. Despite the larger logistics cost and recall cost per unit of product, the logistics advantage of the SPS generates greater positive utility to consumers, which can effectively offset the impact of higher costs on profits. However, when the sinking logistics speed slows down, its advantage gradually weakens. When delivered at the same time as the OPS, sinking logistics not only increases the logistics costs of advanced delivery, but also creates losses due to the uncertainty of the final payment by the consumer. Therefore, if the retailer chooses to adopt the SPS, it should increase its logistics speed to the fastest level, so as to not only increase the perceived utility of this purchase, but also to increase consumers’ favorability and satisfaction with the brand and promote secondary purchase activities.

### 6.4. Retailer’s choice of presale strategy

The above analysis provides relevant support for retailers to choose SPS or OPS under the deposit inflation mode. The detailed analysis results are shown in Table 2. When selecting presale strategies, retailers should consider the consumers’ logistics perception factor *η*,the percentage of consumers paying the final payment for sinking presale *β*, and the time of logistics *l*. When $\eta \geq \frac{\left[1-(\theta -1)k\right]\left[\delta \beta +\gamma \left(1-\beta \right)\right]}{\left(k+\beta -k\beta \theta \right)}$ or $\beta \geq \frac{\gamma \left[1-(\theta -1)k\right]-k}{(1-k\theta )+(\gamma -\delta )\left[1-(\theta -1)k\right]}$, it is more advantageous for retailers to choose SPS. In these situations, the demand for SPS is larger compared to OPS, and the pricing of SPS is always higher than that of OPS, enabling retailers to generate higher profits. However, when opting for SPS, the retailer’s delivery speed must not be lower than or equal to that of OPS. Otherwise, SPS will not positively influence consumer purchase decisions and may even increase the logistics costs associated with early delivery. Therefore, when retailers choose SPS, it is crucial to strive for the fastest possible logistics delivery speed. This approach not only enhances consumers’ perceived value during the shopping experience but also improves their favorability and satisfaction towards the brand, thereby fostering repeat purchases.

**Table 2:** Comparison of two presale strategies.

Variables	Strategies		Results	Conditions
	OPS	SPS		
$p_{a}{}^{*}$	Equations (10)	Equations (22)	$p_{a2}{}^{*}\geq p_{a1}{}^{*}$	—
$D^{*}$	Equations (11)	Equations (23)	$D_{2}{}^{*}\geq D_{1}{}^{*}$	$\eta \geq \frac{\left[1-(\theta -1)k\right]\left[\delta \beta +\gamma \left(1-\beta \right)\right]}{\left(k+\beta -k\beta \theta \right)}$ or $\beta \geq \frac{\gamma \left[1-(\theta -1)k\right]-k}{(1-k\theta )+(\gamma -\delta )\left[1-(\theta -1)k\right]}$
$\pi^{*}$	Equations (12)	Equations (24)	$\pi_{2}{}^{*}\geq \pi_{1}{}^{*}$	$\eta \geq \frac{2l\left[B\left(1-\alpha \right)\left(1-\lambda \right)\lambda h_{2}+B\alpha \lambda p-A\alpha \lambda (p-c_{o})-AE\beta c_{o}\right]-AEG}{BE}$ or $\beta \geq \frac{A\left[\alpha \lambda (p-c_{o})+E\beta c_{o}\right]}{\left(1-k\theta \right)\left[\left(1-\alpha \right)\left(1-\lambda \right)\lambda h_{2}+\alpha \lambda p\right]}-\frac{k}{\left(1-k\theta \right)}$

## 7. Numerical analysis

In the previous section, we compared the two pre-selling strategies and found that optimal demand and optimal profit are somewhat related to individual parameters. In order to more comprehensively study the impact of each parameter on the retailer’s presale strategy, this section uses MATLAB to specifically analyze from three perspectives: consumer behavior, presale deposit and sinking logistics. It specifically analyses the impact of parameters on product presale price, consumer demand and profitability, providing a reliable basis for retailers to optimize their presale strategies.

Based on the previous definition of parameters, the existence condition of the optimal solution and the setting of the initial state parameters in literature [3,4,30], we set the initial setting parameters as follows: N=10000, $h_{1}=100$, $h_{2}=300$, p=200, $c_{0}=100$, α=0.4, β=0.90, λ=0.9. θ=1.25, k=0.2, l=1, η=8, δ=2, γ=3.

### 7.1. Consumer behavior

Consumer behaviors includes the proportion of strategic consumers *α*, the proportion of consumers paying the final price *β* and consumer’s perceived utility coefficient *λ*. To analyze the influence of these parameters on product presale price, demand and profitability, we separately analyze the evolution of $\alpha \in \left(0,1\right)$, $\beta \in \left(0,1\right)$, $\lambda \in \left(0,1\right)$.

**Fig 4:**
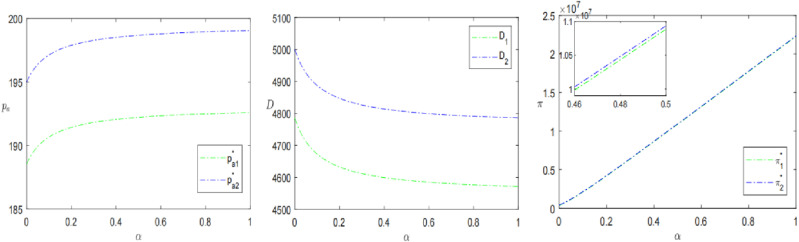
The impact of the proportion of strategic consumers *α* on presale strategies.

As shown in Fig 4, the presale price, demand and profit are all higher when retailers choose the sinking presale strategy. In addition, as the percentage of strategic consumers *α* increases, the presale price and profit increases, but the demand decreases. This also indicates that the proportion of strategic consumers positively affects the presale price and profit but negatively affects the sales of the product. Liu et al. [26] also discussed the relationship between the proportion of strategic consumers and product pricing, sales volume and profit, but the results were opposite to this study. This is because strategic consumers, by comparing the utility of the advance selling period with the regular selling period, find that the negative effect of sinking presale’s price is more significant, which in turn leads to a reduction in demand for the advance selling period. The price sensitivity of short-sighted consumers who are willing to purchase during the presale period is lower than that of strategic consumers. In this scenario, in order to maximize their own interests, retailers will set higher prices to obtain higher profits, leading to an increase in both presale prices and profits. Hence, it can be seen that retailers can stimulate consumers’ short-term shopping desire to a certain extent by alleviating the strategic behavior in shopping, thus effectively boosting consumer demand and expanding market share.

**Fig 5:**
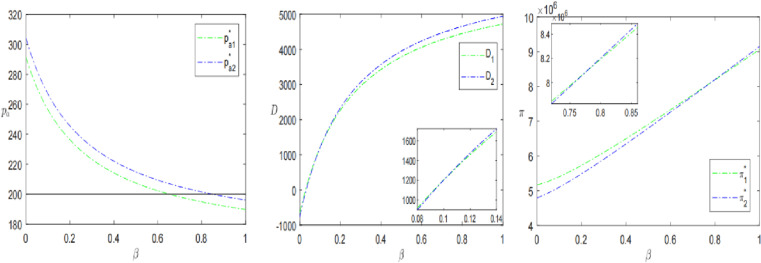
The impact of the percentage of final payment by consumers *β* on presale strategies.

Fig 5 illustrates the effect of the percentage of final payment *β* on the pre-sale price, demand, and profitability. The pre-sale price for the SPS is higher than that for the OPS and $p_{a}$ decreases as *β* increases. In addition, when *β* is small, the consumer demand and profit of the retailer choosing the OPS are greater than that of the SPS. However, as *β* grows larger, the demand and profitability of the SPS gradually increase and the retailer is more inclined to opt for the SPS. This is consistent with Propositions 2 and 3. From the perspective of consumer psychology, the initially high presale price can serve as an “anchor point” that guides their perception of the product’s value. Even if the subsequent price is reduced, consumers still perceive it as a good deal compared to the initial high price, making them more willing to make a purchase. From the retailer’s perspective, profit maximization is their guiding principle, hence the continual adjustment of presale prices to achieve this goal. A higher presale price allows for greater early-stage profits, while adjusting the presale price downward as the final payment proportion increases can attract more consumers, thereby boosting sales and profits. When the proportion of consumers paying the final payment increases to a certain value, the profit under the sinking pre-sale mode exceeds that under the ordinary pre-sale mode. Wang et al.’ s [37] research reached a similar conclusion, but due to consumer demand and parameter setting, it only shows the consistency of the evolution trend.

**Fig 6:**
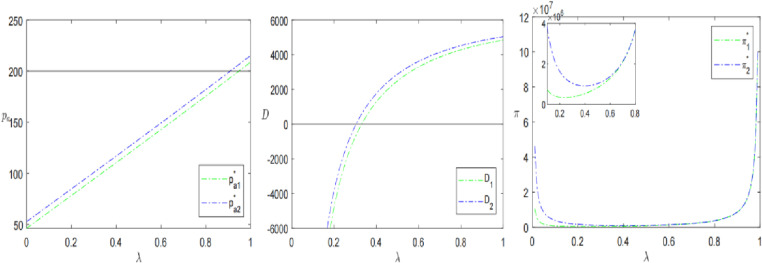
The impact of consumers’ product valuation coefficients *λ* on presale strategies.

The trends of presale price, demand and profit with consumer’s product valuation coefficient *λ* for different presale strategies are shown in Fig 6. Wang et al. [37] involved the expected valuation coefficient of consumers in the study, but did not conduct in-depth research. This study conducted a sensitivity analysis of the valuation coefficient and obtained new findings. The product valuation coefficient due to presale uncertainty plays a positive role in the presale price and consumer demand. And consumer demand does not exist when *λ* is small. In contrast, profits tend to decrease and then increase with the increase of *λ*, reaching the lowest point of profits at λ=0.24, λ=0.40, respectively. Meanwhile, the profit of the SPS is slightly higher than that of the OPS, suggesting that SPS can somewhat mitigate the decrease in product valuation caused by pre-sales uncertainty. It can also be found that the profit is not meaningful when *λ* tends to be 0 or 1, which means that the consumer’s product valuation coefficient is not better to be larger.

### 7.2. Presale deposit

**Fig 7:**
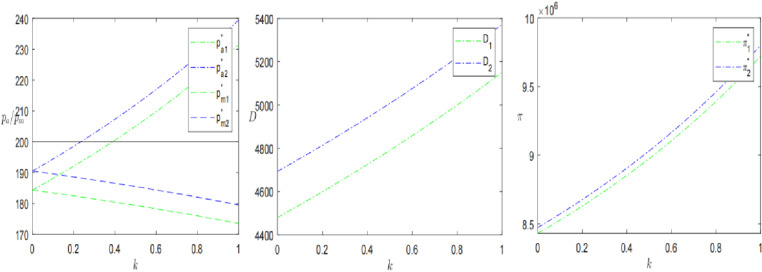
The impact of presale deposit ratio *k* on presale strategies.

**Fig 8:**
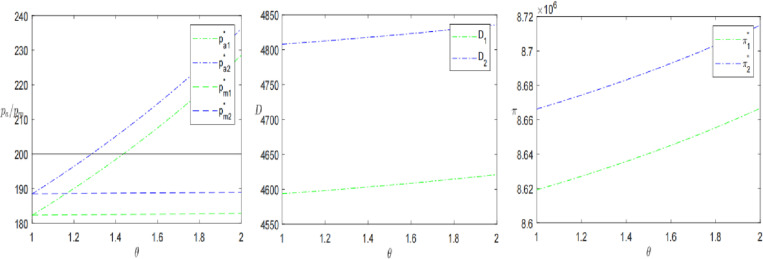
The impact of deposit inflation factor *θ* on presale strategies.

Then, from the perspective of presale deposit, this paper analyzes the relationship between deposit ratio *k* and deposit inflation factor *θ* with presale price, demand and profit. Keeping all other parameters constant, let *k* increase from 0 to 1 and *θ* increase from 1 to 2. The results are shown in Figs 7 and 8.

Fig 7 shows the effect of the presale deposit ratio *k* on presale price, demand and profit for the two presale strategies. It is clear that the presale price $p_{a}{}^{*}$, demand $D^{*}$ and profit $\pi^{*}$ increase with the increase in deposit ratio *k*, while the consumer’s out-of-pocket price $p_{m}{}^{*}$ decreases with the increase in deposit ratio *k*. For the same deposit ratio, the SPS is more advantageous than the OPS. Although the larger the deposit ratio, the larger the profit. But in practice it is not advisable for the retailer to set the deposit amount too large. On the one hand, this is because in the presale form of “pricing + final payment”, the deposit is to lock the product in advance and is usually non-refundable, and most of the product amount should be paid in the final payment period. On the other hand, the market is in a competitive environment. Faced with the same product, consumers will choose a retailer with a smaller deposit to make a purchase, to avoid the valuation losses associated with pre-sale uncertainty. This is why the ratio or amount of deposit is usually determined by the external market. The retailer then adjusts the deposit settings to suit the characteristics of its products. Liu et al. [30] obtained the opposite conclusion from this study. The reason is that although the advance selling price in this study increases with the increase of the deposit ratio, the consumer’s out-of-pocket expenses are decreasing, which in turn increases consumer demand and improves the retailer’s profit.

Fig 8 shows the relationship between the deposit inflation factor *θ* and presale price, paid price, demand, and profit. As the deposit inflation factor increases, the presale price, consumer demand and profit tend to increase for both presale strategies. This is consistent with the trend in deposit ratios described above. Unexpectedly, the consumer’s out-of-pocket price $p_{m}{}^{*}$ always remains the same, regardless of the value of the deposit inflation factor. In addition, when θ>1.24/1.41, the presale price is greater than the in-stock price, but demand and profits are not affected. In other words, the larger the inflation factor is, the larger the presale price is, and the greater the demand and profit are. This indicates that consumers’ purchasing behavior is highly influenced by external discounts, ignoring the actual amount paid. A higher discount can bring more satisfaction to consumers. Therefore, the retailer can make appropriate use of the psychological discount effect of consumers for presale strategies’ optimization, such as increasing the deposit inflation factor, thus promoting demand and profit. From the perspective of alleviating consumers’ strategic waiting behavior, Yin and Wang [31] discussed the impact of deposit expansion coefficient on deposit ratio and spot product pricing, while this study mainly focuses on the impact of expansion coefficient on advance selling price, demand and profit.

### 7.3. Sinking logistics

**Fig 9:**
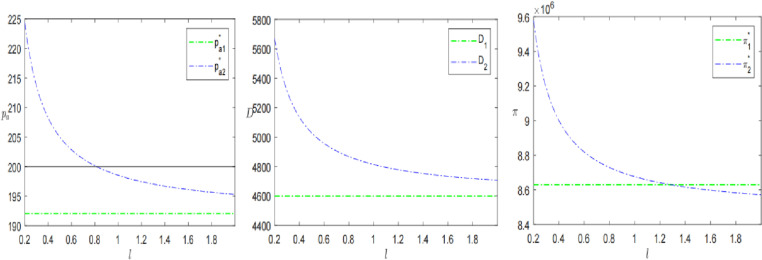
The impact of sinking logistics time *l* on presales strategies.

**Fig 10:**
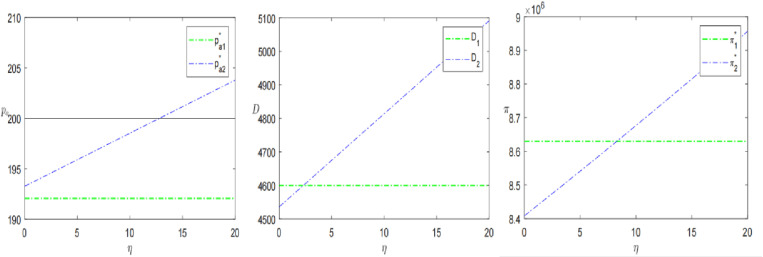
The impact of consumer’s logistics perception coefficient *η* on presale strategies.

Finally, this paper analyses logistics time *l* and logistics perception coefficient *η* to further explore the impact of sinking logistics on presale decisions. The results are shown in Figs 9 and 10.

Keeping all other parameters constant, let logistics speed *l* time from 0.2 to 2. As shown in Fig 9. The presale price, consumer demand, and profit of the SPS all show a decreasing trend with the increase of *l*. Among them, when the logistics speed is faster, the presale price will be higher than the in-stock price, while the demand and profit will be larger instead. This is because the logistical advantage of the SPS not only gives consumers greater utility, making them unwittingly willing to pay more for the purchase, but also stimulates consumers’ purchasing behavior and enhances their willingness to buy. However, when the retailer’s logistics are slow l≥1.29, the profit of the SPS is smaller than that of the OPS. As stated in Proposition 4, a retailer who chooses the SPS should maximize the logistical advantage of the advance selling period and actively promote its advantage, thereby contributing to profit maximization. As previously stated, Zhang et al.’ s research also considers the differences in logistics services between SPS and OPS [3]. When the proportion of knowledge consumers is high, consumers are more inclined to use SPS goods. The proportion of strategic consumers set in this study is 0.4. So, when the logistics time is less than a certain threshold, the retailers using SPS have higher profits. Previous studies support this result. In addition, the study also introduces consumers’ logistics perception and further explores the changes under the influence of different consumers.

Keeping the other parameters constant, let the logistics perception coefficient *η* increase from 0 to 20. As shown in Fig 10, The coefficient has a positive effect on the presale price, demand and profit. As the coefficient increases, the price, demand and profit increase. When η≥12.8, the pre-sale price is higher than the in-stock price. When η≥0.28, the demand of the SPS is larger than that of the OPS. When η≥0.83, the profit of the SPS is than that of the OPS. This indicates that when the logistics perception coefficient is large, it is advantageous for retailers to choose the SPS. Therefore, in the decision-making, the retailer should be aware of the consumer’s perception of logistics in advance. If the logistics perception coefficient is greater than a threshold, it may choose the SPS. Proposition 3 is further validated. Zhang et al. [3] divided consumers into bounded rational consumers (only recognizing the advantages and disadvantages of OPS) and knowledge-based consumers (they can sensitively capture the differences between SPS and OPS, that is, the differences in logistics services). The more knowledge-based consumers, the greater the profit of the e-commerce platform adopting SPS. The above research also supports this result from the side.

## 8. Conclusions

### 8.1. Research results

Based on the theory of consumer utility, this paper considers the strategic behavior of consumers in advance selling, and constructs and discusses the two advance selling strategies of the retailer’s sinking advance selling and ordinary advance selling under the deposit inflation mode. In addition, the effects of various parameters on the pre-sale price, consumer demand and profit are analyzed from the perspectives of consumer behavior, pre-sale margin and sinking logistics, in order to provide some reference for retailers’ pre-sale strategy selection.

First, in the existing SPS research, the consumer’s logistics perception factors are ignored. The study found that when the consumer’s perception of logistics or the percentage of consumers paying for the sunken pre-sale payment exceeds a certain threshold, and the speed of SPS logistics is at least equivalent to that of OPS, the retailer finds that choosing SPS is more advantageous. Moreover, when the consumer ‘s logistics perception coefficient, the proportion of the tail payment, and the logistics speed value of the SPS are higher, the retailer ‘s profit will be greater. This finding aligns with the study by Wang et al. [23], which highlights the importance of time and logistics factors in consumer decision-making.

Second, exploring the OPS and SPS under the deposit expansion mode, it is found that when the retailer adopts SPS, setting a relatively high advance selling price can compensate for the higher cost of logistics sinking and the loss related to consumer purchase uncertainty. However, the deposit amount should not be set too large, otherwise consumers will choose retailers with smaller deposit amounts to buy. This is consistent with the work of Yin and Wang [31], who explored how adjusting pre-sale prices can mitigate the negative impact of uncertainty on consumer behavior. Additionally, the findings by Zhang et al. [3] support our assertion regarding the influence of deposits on consumer purchasing behavior, further confirming the psychological discount effect mentioned in our study.

Third, regardless of the deposit inflation factor, the amount paid for the product remains unchanged. Increasing the deposit expansion coefficient, consumers are affected by the psychological discount effect, ignoring the actual payment price, which in turn increases the market demand and profits. Therefore, retailers can use the consumer discount effect to optimize their pre-sales strategy.

### 8.2. Theoretical and practical implications

In terms of theoretical insights, this paper constructs game models for two presale strategies and investigates optimal presale pricing, consumer demand, and retailer profit under different presale modes, enriching theoretical research on how consumers perceive the value of presale products and make purchasing decisions. Additionally, through a comparative analysis of two presale strategies—SPS and OPS—the study discusses the impact of various parameters on presale pricing, consumer demand, and profit. The research findings provide theoretical support for retailers to make informed decisions when selecting presale strategies.

In practical significance, firstly, understanding consumer behaviors and preferences is crucial for devising successful presale strategies. Retailers should consider factors such as logistics perception, the proportion of consumers making final payments, and sinking presale logistics speed when choosing presale strategies. Secondly, pricing strategies should be carefully considered. When adopting the sinking presale strategy, setting relatively higher presale prices can help offset higher costs and reduce negative impacts from consumer uncertainty in purchasing decisions. However, presale prices should not be set excessively high, as this may drive consumers to choose competitors with lower deposit amounts. Finally, this study emphasizes the importance of considering consumer discount effects when adjusting presale strategies. Regardless of variations in the deposit inflation factor, consumer payment costs remain unchanged, but a higher inflation factor can increase demand and profit. Therefore, retailers can leverage this effect to adjust presale strategies for maximizing profit and effectively participating in market competition.

### 8.3. Limitations and future research directions

This paper explores the impact of retailers’ presale strategy from three perspectives: consumer behavior, presale deposits, and sinking logistics. However, due to the limitations in the problem setting, the effects of consumer refunds and delayed payment times have not been considered. Therefore, these two aspects could be expanded upon in future research. On one hand, return services can increase consumers’ willingness to make purchases, but the associated costs may harm retailers’ interests. Thus, whether to offer refunds when consumers are dissatisfied with presale products can also impact retailers’ strategies. On the other hand, there is a time lag in the “deposit + final payment” model, so the impact of delayed payment times on consumer purchasing behavior needs to be considered. If consumers are satisfied with delayed payments, they are more likely to choose to purchase from the retailer. However, if payment delays are too long, consumers may become dissatisfied and turn to competitors for a faster purchasing experience. Therefore, determining the payment schedule is an interesting and worthwhile area for further study.

## Supporting information

S1 File Data set. (XLSX)
